# Abstract of the Proceedings of the Buffalo Medical Association

**Published:** 1863-07

**Authors:** J. F. Miner

**Affiliations:** Sec’y.


ART. II.—*Abstract of the Proceedings of the Buffalo Medical Association.*

TUESDAY EVENING, June 2, 1863.

Dr. H. M. Congar, President, in the Chair. Minutes of the last meeting read and approved.

*Voted*, that Dr. Charles Winne be a member of the Association upon compliance with the By-Laws.

*Voted*, that more time be allowed the retiring President to prepare address.

*Dr. Lockwood* reported the following case of Aneurism, which had recently been under his observation and treatment: George Lowry, 49 years old, about four years since, while lifting upon his shoulder a quarter of beef, felt something give way, and suffered considerable pain or uneasiness in the upper portion of the chest for a few days, but soon resumed his usual occupation—wholesale butcher. Was however troubled with slight cough, and oppressed breathing, especially in the recumbent position. After a few weeks' attendance at this time, he lost sight of the case for a period of two years, during which time he was treated very attentively by a notorious Homœophathist, as Mr. Lowry informed him, for "*Neuralgic Rheumatism*," after which he again came under his care. Upon examination a tumor presented at the superior portion of the chest, a little to the right of the sternum, apparently somewhat elevating the right clavicle, and distinctly observable above the margin of that bone. Pulse was about 80 per minute and quite natural. Had an anxious countenance; appeared and said he felt as though he had been badly frightened; said "something was wrong, yet he did not feel sick." Upon examining the chest, the heart sounds were natural; respiration was hurried and labored, hardly possible, while in the recumbent position, making it necessary to keep his head and shoulders elevated. At this time the pulsation in the tumor was not very distinct, but soon became much more so. The peculiar *bruit* of aneurism was plainly heard, and the nature of the case no longer obscure or uncertain. The tumefaction gradually increased until it stood out prominently in front and extended upwards so that before death the chin would sometimes rest upon the upper margin of it. The pulsations in the radial arteries of both arms were remarkably unchanged, which fact constitutes one striking peculiarity of the case. Death took place gradually from exhaustion, induced mainly perhaps by the injurious compression which the tumor exerted upon the neighboring organs—trachea and bronchial tubes—and not by sudden rupture of the sac, or the development of inflammation, suppuration or mortification, the more common modes of termination in aneurismal disease.

One other point in the case is worthy of note, viz: the length of time which elapsed after the commencement of the disease, or first rupture of the vessel until its termination. That aneurism of the arch of the aorta should exist for four years is quite remarkable, it being much more common for it to produce death in the space of a few weeks.

Dr. L. was assisted in the *post mortem* examination by Dr. Miner, which was made in presence of Drs. White and Bayless. Dr. Miner had also examined the case during life. Discoloration of the integuments around the tumor was noticable even some days before death, but was still more extensive after. The aneurismal sac was very large, containing by estimation two pounds of fibrinous deposit and coagulated blood. The walls of the tumor appeared to consist only of the condensed tissue which surrounded the vessel, the coats of the artery appearing wholly absent; yet possibly the external tunic of the vessel might have been greatly attenuated and spread over the tumor. The hurried examination would not settle that point; yet it appeared to be wholly absent. As is common in such cases, the pressure had produced erosion of the sternum clavicle and first rib. The tumor appeared to have nearly made its way through the integument, the skin in the most prominent part with a few lines of condensed tissue forming the only covering of the aneurismal sac.

*Dr. Congar* remarked that he had written a small treatise upon the "Laws of Hereditary Descent," and that he would like to read a portion of it for the purpose of obtaining the unbiased opinion of the Association.

*Voted*, that at a special meeting, Dr. Congar be invited to read his paper.

*Dr. Rochester* introduced the subject of, poison contained in articles sold for "Insect Powder," and spoke of the danger attending the use of such articles; mentioned the instance of one man who made and sold in Buffalo great quantities of arsenite of copper.

Other members of the Society spoke also of the danger, and also of the propriety of guarding the public from injury.

*Voted*, that the Secretary be requested to publish in the daily papers the composition of some of these compounds and the danger attending their use.

*Dr. White* said he had been making use of solution of persulphate of iron in chronic cystitis, and though it was difficult to tell how much was due to its influence and how much to other causes, still it seemed to have been beneficial in some cases which he related. One patient recovered rapidly and was soon well. Another gentleman who was feeble and had to be carried upon a bed from the depot to his boarding house, took 15 drops every four hours, properly diluted; had a blister applied to the perineum, and opium suppository to relieve pain. He was soon very much better; deposit in the urine much less, micturition less frequent, strength improved so that he walks out, and says he feels quite well. How much is due to the iron and how much to other causes he could not determine. That the iron was an astringent tonic, giving tone to the system there could be no doubt. Perhaps others had used it for this disease, and yet he had not heard of it, and had mentioned his success not as anything original, but thinking it was useful, and that others might like to make trial of it.

*Dr. Cronyn* related the case of a young man who was struck by a brick falling upon the top of the head near the center. There was at the time of injury evidence of depression sufficient to make it probably necessary to trephine. At the expiration of 24 hours consciousness was restored, and with a return of sensibility *thirst* commenced, which was very great with dry skin, and indications of diabetes, with loss of 24 pounds in weight in one month. He had carefully tested the urine for sugar, but had discovered nothing.

The patient was placed upon quinine, opium and iron, and had improved considerably. Complains still of great weakness. Did not regard the case as diabetes, but rather as a functional disease induced by injury of the nervous centre. Thought it indicated irritation of the fourth ventricle, that being supposed to have some influence over the urinary secretion, thirst, &c., &c. The case was a remarkable one, and presented many points of great interest, both pathologically and therapeutically.

*Dr. Rochester* related a case which at first he regarded as concussion, but afterwards it seemed to be marasmus, induced probably by the concussion. Related the case only as showing that concussion might produce change in the nervous system and perverted function.

*Dr. Peters* related a case seen in Emory Hospital, Washington, where a man was found who was shot at the last battle of Bull Run, the ball having passed directly through the top of the head, leaving a large opening, both at point of entrance and exit of the ball. The second day after admission to Hospital he was found sitting in a chair reading a newspaper.

*Dr. White* related a case of vesico-vulval fistula, as the result of laceration in labor. Patient was 19 years old, prima para, and delivered with forceps. The opening was above and to the right of the urethra. This was a new site for fistula, though vesico-vaginal fistula was common after severe protracted labors, and instrumental deliveries. He had made operation for the cure of it; and the next day after thought it was leaking, but since that it seems to be doing well and likely to be entirely successful.

*Drs. Lockwood, Rochester and Miner,* spoke of the prevalence of measles, and also another eruptive disease which had been regarded as *rubeola sine catarrho* by some physicians, while others called it roseola. Dr. Rochester did not regard it as rubeola at all, and related cases where it, and true measles was very nearly associated, measles occurring the fourth day after the disappearance of roseola. Knew of children who had roseola and who had previously had measles. Distinguishes the diseases by the mildness of the symptoms and by the eruption being punctate, less elevated than measles, and disappearing much sooner. Dr. Lockwood had seen a great many cases and had previously regarded it as measles, but had more recently entertained doubts about it. Dr. Miner spoke of the disease as appearing to be equally as contagious as measles, to be communicated in the same manner, and in some cases to be quite as severe as rubeola in mild form. Could only repeat what he had before said, that roseola was universally regarded as a non-contagious, non-infectious disease, so that unless we repudiate the usual definition given of roseola, we cannot regard it as that disease, not if we admit its contagion, which seems to be conceded by all. Is this disease contagious? if so, it is not roseola. If roseola, then roseola is a contagious disease. Thought it sufficiently answered in all respects the description given of *rubeola sine catarrho* to be the disease so named by authors, which is said to often occur with true measles and not to prevent its appearance, or protect the system from its attack in the least degree.

After the transaction of some legislative and miscellaneous business. *Voted* to adjourn. J. F. MINER, *Sec'y.*

